# Epilation of eyelashes

**Published:** 2008-12

**Authors:** Sue Stevens

**Affiliations:** Nurse Advisor, *Community Eye Health Journal*, International Centre for Eye Health, London School of Hygiene and Tropical Medicine, Keppel Street, London WC1E 7HT, UK.

**Figure:**
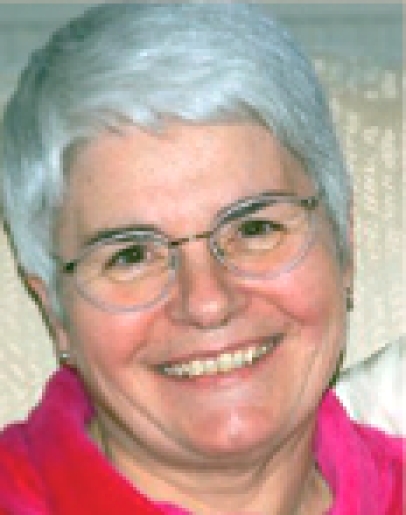


## Remember!

**Before performing any eye procedure:**

- Wash your hands (and afterwards too)
- Position the patient comfortably with his/her head supported
- Avoid distraction for yourself and the patient
- Ensure good lighting
- Always explain to the patient what you are going to do.

### Indications

- To remove ingrowing eyelashes (trichiasis)
- To prevent corneal abrasion

### You will need

- Magnification (magnifying loupe)
- Torch or flashlight
- Gauze swabs
- Local anaesthetic eye drops
- Epilation forceps
- A helper

### Preparation

- Explain the procedure and advise the patient that it will cause some very brief discomfort
- The patient, helper, and examiner should be positioned appropriately. The helper can hold the torch (Figure 1).
  **Figure 1:**
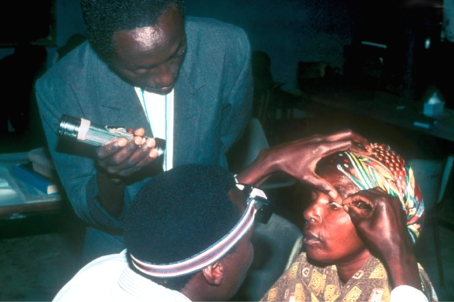

### Method

- Instil the local anaesthetic eye drops
- Using magnification, identify the eyelashes which need epilating (Figure 2)
  **Figure 2:**
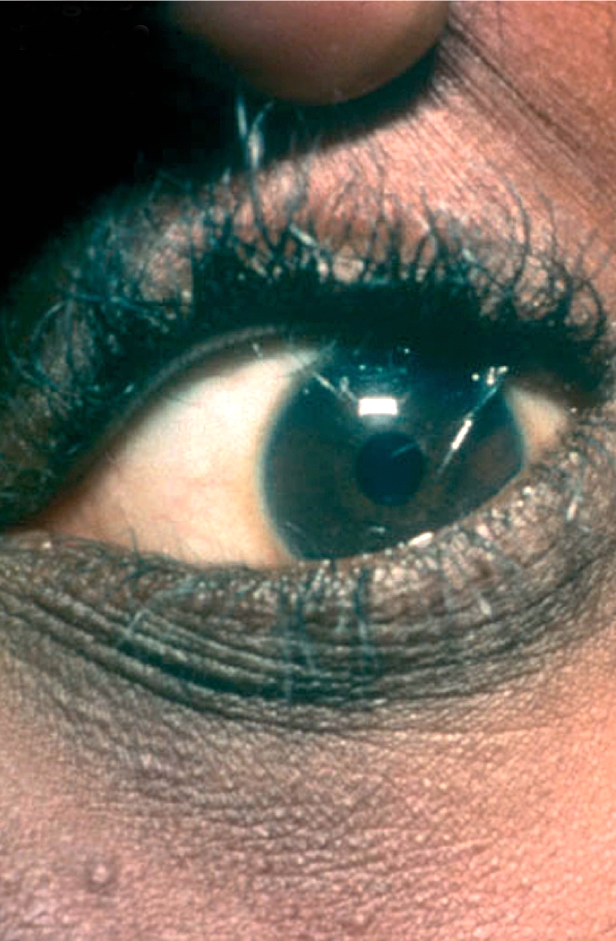
  **For lower eyelashes**:
  - ask the patient to look up, fix his/her gaze, and keep still
  - with an index finger, gently hold down the lower eyelid
  **For upper eyelashes**:
  - ask the patient to look down, fix his/her gaze, and keep still
  - with a thumb, gently ease the upper eyelid up against the orbital rim
- With the epilation forceps in the other hand, hold the ingrowing eyelash close to its base and pull gently forward to pluck it out (Figure 3)
  **Figure 3:**
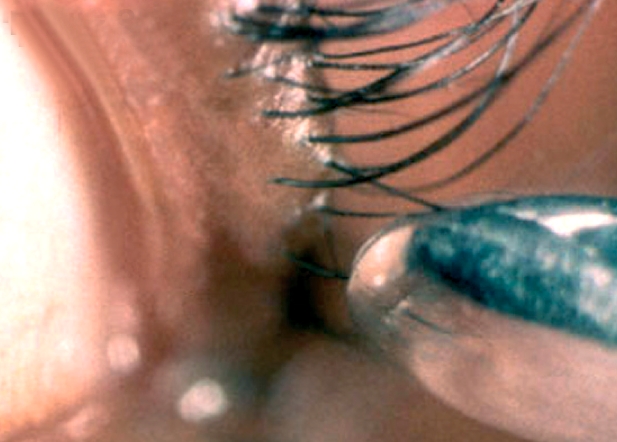
- Repeat until all ingrowing lashes are epilated
- Between each epilation, wipe the eyelash off the forceps with a clean swab (Figure 4)
  **Figure 4:**
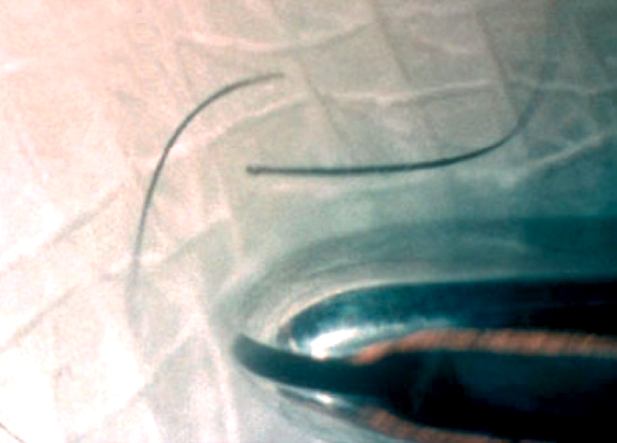
- Reassure the patient when all ingrowing lashes have been removed and advise him/her not to rub the eye.

